# Comparison of femtosecond laser-assisted corneal intrastromal xenotransplantation and the allotransplantation in rhesus monkeys

**DOI:** 10.1186/s12886-017-0595-z

**Published:** 2017-11-09

**Authors:** He Jin, Liangping Liu, Hui Ding, Miao He, Chi Zhang, Xingwu Zhong

**Affiliations:** 1 State Key Laboratory of Ophthalmology, Zhongshan Ophthalmic Center, Sun Yat-sen University, Guangzhou, 510060 China; 2 Hainan Eye Hospital, Hainan Key Laboratory of Ophthalmology, Zhongshan Ophthalmic Center, Sun Yat-sen University, Haikou, 570000 China

**Keywords:** Femtosecond laser, Xenotransplantation, Rhesus monkey, Glycerol, SMILE

## Abstract

**Background:**

In our previous study, we showed that both allogeneic and autogeneic small-incision femtosecond laser-assisted corneal intrastromal transplantation are safe and effective surgeries. However, the results of small-incision femtosecond laser-assisted intrastromal xenotransplantation have not yet been explored. Additionally, we suggest that glycerol-dehydrated corneal lamellae might provide a possible alternative for this xenogenic implantation approach.

**Methods:**

Corneal inlay lamellae were produced from rabbits and humans using femtosecond laser-assisted surgeries and were dehydrated in glycerol for 1 week at 4 °C. These xenogeneic glycerol-dehydrated grafts and fresh allogeneic monkey lamellae were then implanted into rhesus monkeys using small-incision femtosecond laser assistance. Postoperatively, clinical examinations, AS-OCT measurements and tear inflammatory mediator assays were performed.

**Results:**

There were no significant changes in the transparency of the corneal lamellae after glycerol dehydration. Following implantation, no evidence of tissue rejection or severe inflammatory responses was observed in the monkeys, and the host corneas remained transparent throughout a 6-month observation period. The grafts were clearly visible via AS-OCT. Corneal thickness increased 1 week postoperatively but subsequently declined and remained unchanged 1 month after surgery. Significant changes were observed in all tear inflammatory mediators in the ‘Rabbit to Monkey’ group. The trends in changes of tear inflammatory mediators in the ‘Human to Monkey’ group were similar to those in the ‘Rabbit to Monkey’ group. At 1 month post-surgery, the levels of most tear inflammatory mediators had decreased, with the exception of IL-1β, TGF-β1 and IFN-γ in the allotransplantation group.

**Conclusion:**

Small-incision femtosecond laser-assisted intrastromal transplantation minimized invasiveness and improved surgical efficiency. In addition, the host cornea maintained a high level of biocompatibility. Glycerol-dehydrated corneal lamellae might be potentially useful as an alternative inlay xenogeneic material. In this study, we also describe a new treatment that can be used in keratoconus, corneal ectasia, presbyopia, hyperpresbyopia and other diseases.

## Background

Both corneal diseases and injuries can damage the cornea, eventually leading to blindness [1, 2]. The main treatment for corneal damage is corneal transplantation. However, two major problems restrict the clinical application of transplantation. These include severe postoperative complications that lead to graft rejection and the insufficient supply of donor corneas [3].

Penetrating keratoplasty (PKP) was the main transplantation surgery that was used in the 1980s [4]. Postoperative immune rejection is a severely complication that can follow PKP and that can lead to graft endothelial dysfunction and to the long-term survival rate decreasing [4]. The graft survival rates at 1, 3, 5 and 10 years were 84.0%, 69.3, 60.7% and 59%, respectively [5, 6]. The most common cause of graft failure was immunologic rejection. It was followed by late endothelial failure and ocular surface complications, which collectively accounting for over 74% of all the failure cases [6]. However, in recent years, in cases in which corneal disease does not involve the endothelium, the lamellar technique, which produces better clinical results, has rapidly replaced penetrating grafts. This technique aims to selectively replace diseased corneal stromal tissues to minimize the unnecessary replacement of the unaffected healthy endothelial layer [7]. Nonetheless, lamellar keratoplasty (LK) is difficult and time-consuming, and 2.3–3.5% of cases necessitate conversion to PKP intraoperatively [7]. The rate of rejection following LK is lower than that following PKP according to a 5-year follow-up study by Zhang et al. However, similar to PKP, visually limiting factors also include corneal surface distortion, which is created by suture tension, and graft-host interface disparities [8, 9]. The current technique resembles inlay LK, which was first described by Barraquer [10]. Many attempts to perform inlay LK have reduced corneal invasiveness and the influence of the corneal epithelium and endothelium, but previous inlay LK models are limited by the complex nature of the operation, unsatisfactory implantation materials, and graft rejection [10].

In our previous study [11], we found that rhesus monkeys did not suffer corneal rejection after undergoing femtosecond laser-assisted inlay LK with either autotransplantation or allotransplantation. Additionally, a corneal lamellae inlay model avoids stimulating the ocular surface and endothelium and will prolongs graft survival rates to some extent [11]. Furthermore, femtosecond laser cutting is a highly accurate, safe and predictable technique that represents a new choice for corneal refractive surgery and corneal transplantation [12]. Femtosecond laser cutting always achieves a consistent depth and thickness on every occasion inside the corneal stroma and precisely matches donor and recipient tissues [13]. Compared with common LK, femtosecond laser cutting provides the most substantial advantage because it allows a high level of control that largely minimizes its surgical invasiveness.

Another clinical difficulty in corneal transplantation is the insufficient supply of donors. The use of xenogeneic donors may potentially alleviate the shortage of donor corneas, which can be accomplished due to the unique characteristics of the cornea compared with those of other tissues and organs [14]. A recent study has indicated that grafts with low antigenicity can be used as supporting materials for corneal tissue regeneration [15]. The technique of performing corneal dehydration in glycerol was first described by JN McNair [16]. Glycerol dehydration has been shown to substantially decrease the cellularity and antigenicity of donor corneas, particularly in the stroma [17–19]. Thus, glycerol-dehydrated corneal lamellae may provide a possible alternative source of inlay xenogeneic grafts in theory. This is currently a nascent area of study.

We developed a novel small-incision femtosecond laser-assisted surgical approach to reduce surgical invasiveness and improve the ability to match graft and donor tissues. We used femtosecond laser-assisted surgery to form inlay grafts, and we dehydrated xenogeneic grafts in glycerol to reduce their antigenicity. This study was designed to test biocompatibility and to evaluate the effectiveness of small-incision femtosecond laser-assisted surgery when xenogeneic corneal lamellae and fresh allogenic lamellae were implanted in rhesus monkey corneas. We also assessed the biocompatibility of glycerol-dehydrated corneal lamellar as the corneal inlay.

## Methods

### Study design

Corneal lamellae were obtained from three species (humans, rabbits and monkeys) using femtosecond laser-assisted surgery. Two of the xenogeneic grafts types (human and rabbits) were dehydrated in glycerol for 1 week. All grafts were inlayed into rhesus monkey corneas using small incision lenticule extraction (SMILE)-assisted surgery. Four monkeys that received fresh allotransplantation grafts were used as the control group. Another 4 monkeys received human glycerol-preserved grafts and served as the ‘human to monkey’ group (‘H to M’ group). The remaining 4 monkeys received rabbit glycerol-preserved grafts and served as the ‘rabbit to monkey’ group (‘R to M’ group). After surgery, we observed the ocular appearance of the monkeys every day and examined the 12 monkeys that received grafts at 1 week and 1, 3 and 6 months after implantation.

### Animals

All experimental procedures were approved by the Ethics Committee of Hainan Eye Hospital of the Zhongshan Ophthalmic Center, Sun Yat-sen University (Acceptance number: 2015–010). Five adult New Zealand white rabbits and sixteen 5-year-old rhesus monkeys were used in this study. All animals were housed in clean, environmentally controlled rooms in an Accreditation of Laboratory Animal Care-accredited facility (Animal Experiment Center of the Zhongshan Ophthalmic Center). In addition, the animals were housed individually and provided free access to food and water throughout the study.

### Formation of rhesus monkey allotransplantation grafts

The four rhesus monkeys selected for these experiments were anaesthetized using an intramuscular injection of Zoletil 50 (Virbac, Carros, France, 5 mg/kg) and then underwent SMILE surgery on one randomly selected eye with a myopic treatment correction of −4.00 diopters (D). The femtosecond laser parameters were set as follows: 135 nJ, 120-μm cap thickness, and 7.5-mm cap diameter. A small incision (3 mm) was made at the 140° position with a side cut angle of 90°. Corneal lamellae (81-μm thickness) were isolated for use as fresh allotransplantation grafts.

### Formation of human corneal lamellae grafts

Ten lenticules were collected from 10 people who underwent SMILE surgeries. The spherical equivalent of the patients ranged from −6.00 to −9.00 D. All patients provided written consent to participate in this study. All procedures were approved by the Ethics Committee of Hainan Eye Hospital of the Zhongshan Ophthalmic Center (Sun Yat-sen University) and were performed in compliance with the tenets of the Declaration of Helsinki and with ethics committee approval (Acceptance number: 2015–010).

All surgical procedures were performed by one surgeon (Xingwu Zhong). The surgeries were performed using a VisuMax femtosecond laser system (VisuMax; Carl Zeiss Meditec, Jena, Germany) with a 500-kHz repetition rate. The femtosecond laser parameters were as follows: 135 nJ, 120-μm cap thickness, and 7.5-mm cap diameter. Human corneal lamellae were extracted through an incision (2.5 mm).

The corneal lamellae were immersed in sterile glycerol and dehydrated at 4 °C for 1 week. In addition, they could then be used as human corneal lamellae grafts.

### Formation of rabbit corneal lamellae grafts

**Fig. 1:**
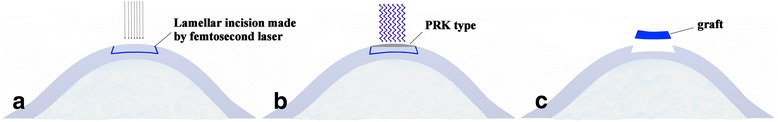
The surgical procedure. **a** Make a lamellar incision (**b**) Cut partial stroma under PRK type (**c**) Separate the lamellar cornea from the stromal bed and be used as the graft

Rabbits were sacrificed using an overdose of anaesthetics (Zoletil 50, Virbac, 50 mg/kg intramuscular injection), and then enucleated. Next, a lamellar incision in the rabbit cornea was made using treatment mode femtosecond laser-assisted LKP (Carl Zeiss Meditec) (Fig. 1a). A small-sized curved interface cone was docked on the center of the cornea. The following surgical parameters were as follows: the diameter of the incision was 7.5 mm; the incision was located at a depth of 320 μm; and the side cut angle was 90°. Next, we performed a photorefractive keratectomy (PRK, Wavelight GmbH, Erlangen, Germany) to achieve a myopic correction of −4.00 D (69.59 mm) (Fig. 1b). We then separated the corneal lamellae from the stromal bed (Fig. 1c).

The corneal lamellae were immersed in sterile glycerol and dehydrated at 4 °C for 1 week before they were used as rabbit corneal lamellae grafts.

### Femtosecond laser-assisted intrastromal implantation

**Fig. 2:**
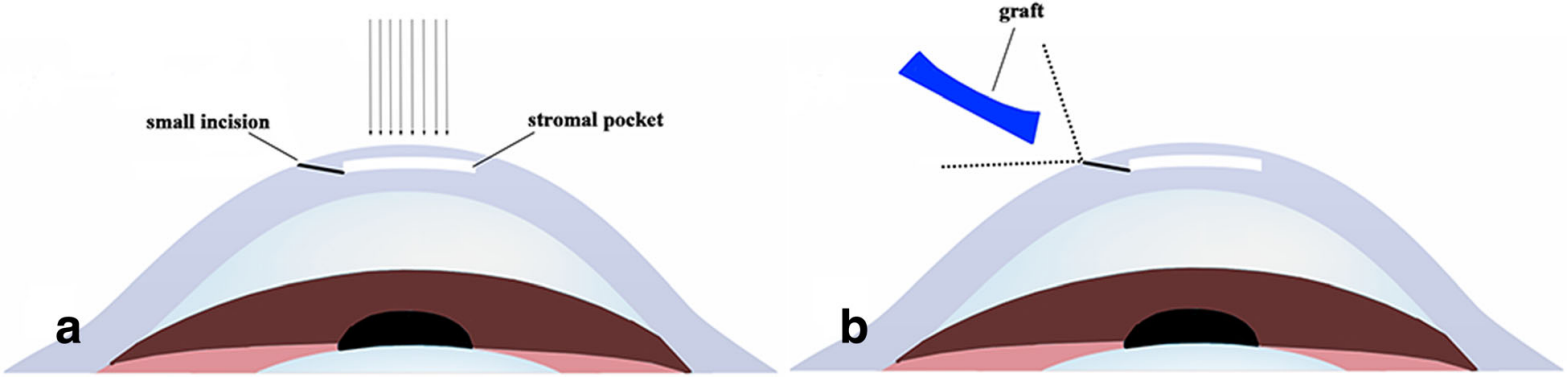
The procedure of implantation. **a** With SMILE surgery assisted, spread the lenticule out, producing a space like a ‘stromal pocket’. **b** Use forceps to implant the graft to the ‘stromal pocket’

Twelve rhesus monkeys were anaesthetized using an intramuscular injection of Zoletil 50 (5 mg/kg, Virbac). They then underwent SMILE surgery in one randomly selected eye to achieve a myopic treatment correction of −0.75 D. The following femtosecond laser parameters were used: 135 nJ, 160-μm cap thickness, 7.8-mm cap diameter, and 7.8-mm lamellar cornea diameter. A small incision was made at the 140° position with a side cut angle of 90°. The lenticule (28-μm thickness) was separated, and a space similar to a “stromal pocket” was produced (Fig. 2a). The graft was implanted in the “stromal pocket” (Fig. 2b). We avoided inverting the surface of the graft during implantation.

Following the surgery, all monkeys received tobramycin and dexamethasone (Alcon Laboratories, US) eye drops 4 times per day for 1 week. We observed the ocular appearance of the monkeys every day and examined the 12 monkeys that received grafts at 1 week after implantation and 1, 3 and 6 months thereafter.

### Clinical examinations

Ocular examinations were followed by a slit-lamp examination to assess changes in implant colour, surrounding host corneal transparency, neovascularization, corneal edema, rejection reactions and implant degradation.

The characteristics of the anterior segment of the eye, including corneal thickness, were assessed using anterior segment optical coherence tomography (AS-OCT, DRI Triton OCT, Topcon Corporation, JP). The central corneal thickness of the rhesus monkey donors was approximately 450 μm, as measured by AS-OCT preoperatively. In adherence with the guidelines of the Animal Care committee (Animal Experiment Center of the Zhongshan Ophthalmic Center), we used a graft thickness of 80 μm to achieve an approximate residual stromal thickness (RST) of 250 μm with the aim of stabilizing the monkeys’ intraocular pressure. Because the thicknesses of the grafts obtained from the human donors were different, we first calculated the predicted change in central corneal thickness (PCT) and compared it to the factual observed change in central corneal thickness (FCT). In the allotransplantation group, the predicted change in this value was 81–28 = 53 μm. The predicted change of the value in the ‘H to M’ group was X-28 μm (where X represents the thickness of the human donor’s lenticule). The thickness of the epithelial layer of the rabbit cornea was approximately 60 μm [20], and the predicted change of this value was 320–60–69.59-28 = 162.41 μm. The FCT was determined by comparing the factual observed central corneal thickness to the pre-operation’s. Statistical analysis was performed using the following formula: △Thickness = FCT – PCT.

### Tear collection and inflammatory mediators assay

Non-stimulated tear samples were collected using disposable 10-μl pipette tips. Tear collection was performed at the inferior marginal region, conjunctiva or lid margin. The levels of interleukin-1β (IL-1β), IL-6, IL-17, transforming growth factor-β1 (TGF-β1), tumour necrosis factor-α (TNF-α), and interferon-γ (IFN-γ) in the collected tears were measured using a Quantibody Human Inflammation Array kit (RayBiotech, Inc., Norcross, GA) according to the manufacturer’s instructions. The signals were captured by a GenePix 4000B microarray scanner (Bio-Rad Laboratories, Hercules, CA) and analyzed with Quantibody Q-Analyzer software (RayBiotech, Inc.).

### Statistical analysis

All statistical analyses were conducted using *SPSS 16.0* statistical software. The data for corneal △Thickness, tear collection and inflammatory mediators assays were recorded as the $\overline{\mathrm{x}}\pm s$ from five separate experiments. The experiments were performed using a completely randomized design. The data were assessed using *one-way ANOVA*. For pairwise comparisons of groups, we used either the *least significance difference test* (*LSD-t*) or *Tamhane’s T2*. *P* < 0.05 was considered significant.

## Results

### Physical characterization of corneal lamellae

**Fig. 3:**
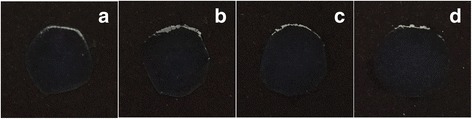
Representative images of corneal lamellae. Though glycerol dehydration, human corneal lamellae (**b**) and rabbit corneal lamellae (**d**) still stayed same transparency compare to the human native one (**a**) and the rabbit native one (**c**)

Gross observations revealed that all cornea lamellae remained transparent after glycerol dehydration (Fig. 3).

### Clinical observation

**Fig. 4:**
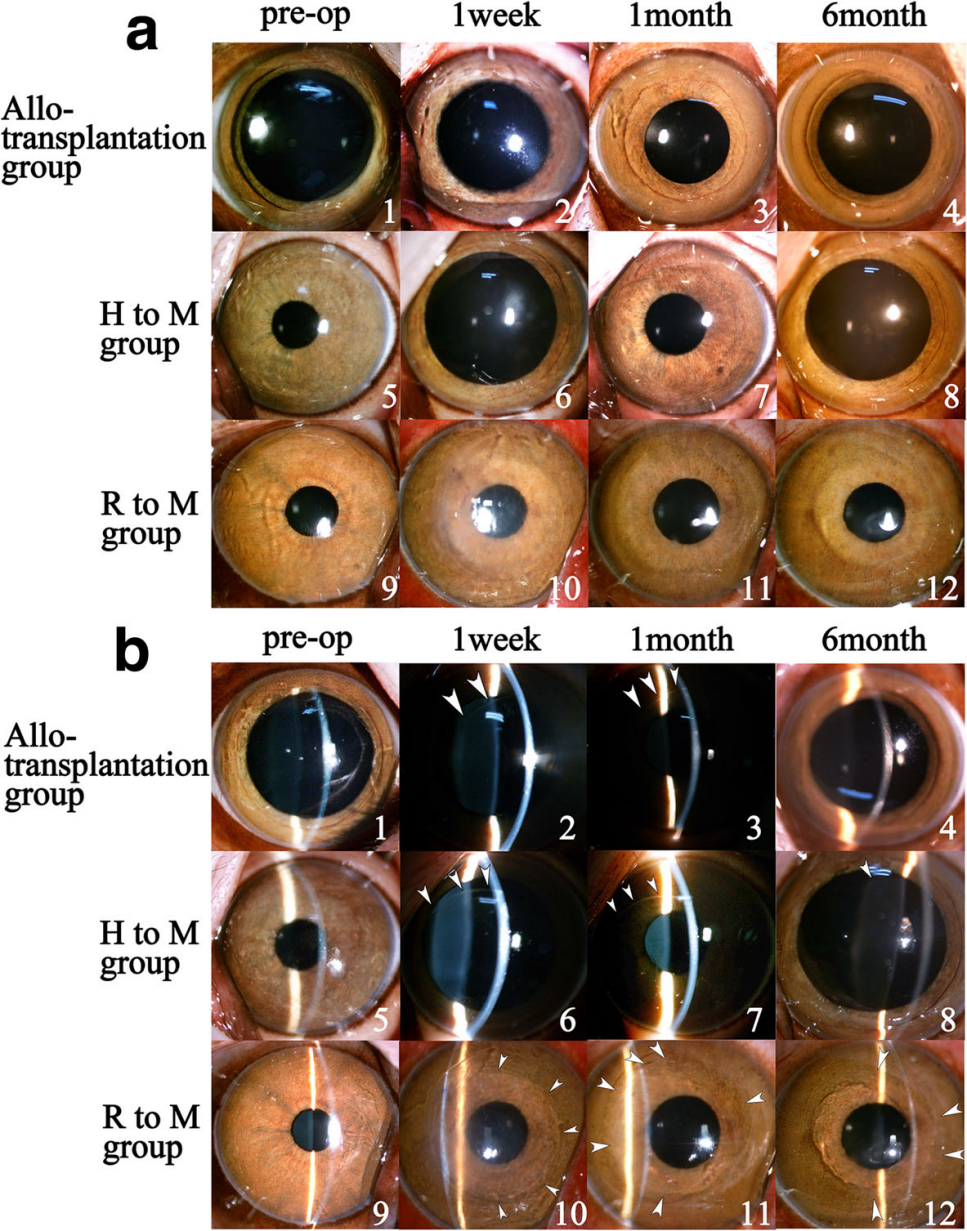
Slit lamp biomicrographs. Preoperative and postoperative ocular observation of rhesus monkeys. After 6 months of surgery, the host cornea exhibited high level of tolerance of the graft, and the graft remained high level of the clarity (**a**). Under retroillumination observation (**b**), at 6-month post-surgery, the cornea remained clear with no haze, no detectable reaction in anterior chamber or any other adverse response. The boundary of the graft became undetected after 6 months of surgery in all groups. The boundary of the graft was marked by white arrows

Throughout the 6-month observation period, all rhesus monkeys in both the allotransplantation group and the two xenotransplantation groups survived without infectious or haemorrhagic complications (Fig. 4a). No obvious eyelid spasm, increased conjunctival redness, corneal neovascularization, signs of graft degradation, or any detectable reaction in the anterior chamber (Fig. 4b) were observed within 6 months after implantations. The host corneal tissue remained transparent.

### AS-OCT observation

**Fig. 5:**
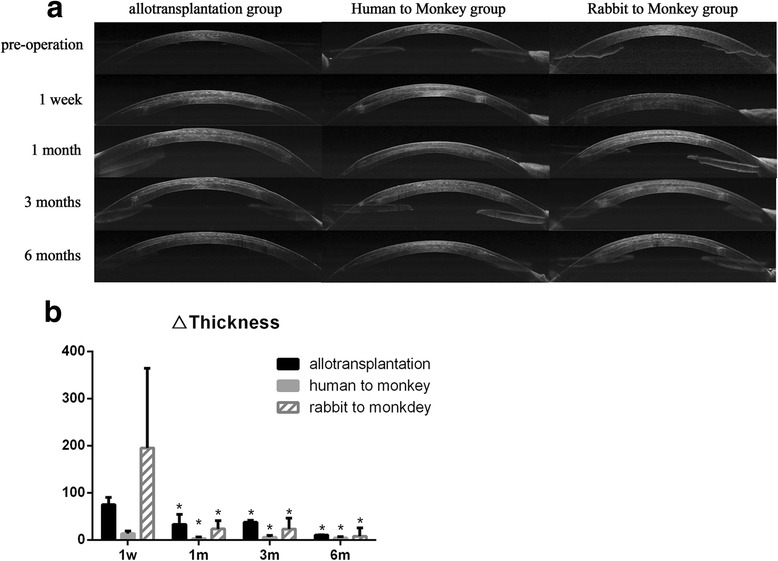
AS-OCT observation. **a** The corneal shape was stable throughout the 6-month observation period, and no graft discharge was observed. **b** Corneal thickness was increased after implantation. One week after surgery, the corneal thickness of the three groups was increased, and the corneal thickness of the‘R to M’ group exhibited the most obvious increase. Central corneal thickness declined after 1 week of surgery, and there was no significant difference in △thickness at 1-month post-surgery. The △thickness values are expressed as the mean ± SD (standard deviation). Statistically significant differences are compared with the 1 week postoperatively and indicated by *P < 0.05. (Allotranplantation group: *P*1 m = 0.005, *P*3 m = 0.009, *P*6m < 0.001; Human to Monkey group: *P*1 m = 0.002, *P*3 m = 0.014, *P*6m = 0.005; Rabbit to Monkey group: *P*1 m = 0.011, *P*3 m = 0.015, *P*6m = 0.009)

In all groups, the grafts were clearly visible in the corneal stroma after implantation. The grafts remained stable within the ‘stromal pocket’ throughout the 6-month observation period (Fig. 5a). Postoperatively, corneal thickness was higher at 1 week, but the thickness of the graft was not significantly altered at 1 month after surgery (Fig. 5b).

### Tear inflammatory mediators

For the inflammatory mediator assays, postoperative values were compared to preoperative values. All tear inflammatory mediators were significantly altered in the ‘Rabbit to Monkey’ group. The trends in changes of tear inflammatory mediators in the ‘Human to Monkey’ group were similar to those in the ‘Rabbit to Monkey’ group. At 1 month after surgery, the levels of most tear inflammatory mediators were lower with the exception of IL-1β, TGF-β1 and IFN-γ in the allotransplantation group (Table 1).

**Table 1:** Time-dependent changes in the tear inflammatory mediators after implantation

	1w	1 m	*P*-value	3 m	*P*-value
	△IL-1β				
Allotransplantation	0.112 ± 0.008	0.111 ± 0.016	0.999	0.700 ± 1.212	0.343
Human to Monkey	47.367 ± 6.208	2.867 ± 0.252^*^	< 0.001	2.700 ± 0.721^*^	< 0.001
Rabbit to Monkey	23.548 ± 17.142	6.043 ± 2.042^*^	0.048	2.836 ± 1.806^*^	0.013
	△IL-17				
Allotransplantation	2.375 ± 4.750	8.133 ± 4.087	0. 694	48.467 ± 56.687^*^	0.036
Human to Monkey	2.552 ± 2.095	0.445 ± 0.516	0.198	20.725 ± 0.106^*^	< 0.001
Rabbit to Monkey	255.292 ± 118.429	13.352 ± 8.128^*^	< 0.001	2.773 ± 3.084^*^	< 0.001
	△IL-6				
Allotransplantation	14.568 ± 3.698	20.096 ± 13.735	0.343	2.167 ± 0.764^*^	0.040
Human to Monkey	15.856 ± 3.782	0.793 ± 0.777^*^	0.002	2.534 ± 5.067^*^	0.003
Rabbit to Monkey	176.301 ± 165.750	39.126 ± 29.103^*^	0.010	8.909 ± 7.047^*^	0.006
	△TGF-β1				
Allotransplantation	467.560 ± 119.913	367.343 ± 232.792	0.960	176.276 ± 51.402	0.384
Human to Monkey	430.024 ± 32.929	134.808 ± 90.996^*^	0.009	189.715 ± 86.430^*^	0.032
Rabbit to Monkey	2853.445 ± 817.661	156.937 ± 36.585^*^	< 0.001	49.067 ± 8.359^*^	< 0.001
	△TNF-α				
Allotransplantation	−12.052 ± 72.799	122.030 ± 65.884^*^	0.011	−52.343 ± 6.073	0.372
Human to Monkey	−65.852 ± 23.792	52.223 ± 15.819^*^	<0.001	−27.287 ± 3.484^*^	0.025
Rabbit to Monkey	−17.137 ± 3.357	−72.716 ± 11.581^*^	0.015	−90.597 ± 25.805^*^	0.002
	△IFN-α				
Allotransplantation	−16.527 ± 14.725	0.820 ± 0.743	0.062	−7.163 ± 6.423	0.263
Human to Monkey	−90.817 ± 5.225	−143.983 ± 4.345^*^	< 0.001	−142.398 ± 4.541^*^	< 0.001
Rabbit to Monkey	−117.396 ± 58.082	−248.080 ± 62.567^*^	0.035	−250.073 ± 82.095^*^	0.023

Statistically significant differences are compared with the 1 week postoperatively and indicated by *P < 0.05

## Discussion

In the present study, we used primates to assess the post-surgical responses of the host cornea to corneal grafts processed from different species. We explored the use of glycerol-dehydrated corneal lamellae as xenogeneic grafts. This is a relatively novel approach to this surgery. We observed no corneal implant rejection or severe inflammatory responses within a 6-month period following the surgery in any of the animal implantation models. These findings suggest that small-incision femtosecond laser-assisted corneal intrastromal implantation can be used to substantially reduce surgical invasiveness and to improve biocompatibility with the host cornea. Furthermore, glycerol-dehydrated corneal lamellae might be a potential inlay xenogeneic material.

Various reports have indicated that glycerol-dehydrated donor corneas can effectively substitute fresh corneas in patch grafts [21]. Storing corneal tissues in anhydrous glycerol at 4 °C has been shown to be as effective as storing these tissues at sub-zero temperatures [22]. Glycerol is a dehydrating agent with antimicrobial and antiprotease properties that also helps to maintain corneal structure [23, 24]. When the structure of the cornea is irreversibly changed, its transparency cannot be recovered [25, 26]. After glycerol dehydration, the corneal lamellae remained transparent. This finding indicates that the structure of the corneal lamellae was not irreversibly altered by this method of dehydration (4 °C for 1 week). Furthermore, glycerol dehydration has been reported to minimise the chances of immune rejection [19]. Therefore, xenogenic corneal lamellae could theoretically be used as xenogeneic inlay grafts.

Known as an “all-in-one” technique, the femtosecond laser-assisted surgeries are safer, more effective, and more precise than other surgical techniques, especially SMILE techniques [27, 28]. Compared with common inlay LK, small-incision femtosecond laser-assisted implantation does not require the use of a blade or any sutures [25]. In addition, corneal surface invasiveness is minimised by the smaller incision (2.5 mm). Thus, small-incision femtosecond laser-assisted implantation carries a lower risk of astigmatism than the usual methods and avoids the induction of most inflammatory mediators in the aqueous humour and tears [29]. During this study, throughout the 6-month period, all monkeys survived and exhibited no implant rejection or adverse complications. The corneas briefly exhibited edema but remained transparent. The corneal epithelium and endothelium were healthy, and the tear film over the cornea was maintained throughout the study. These results indicate that femtosecond laser-assisted minimally invasive surgery could shorten corneal recovery times. Tobramycin and dexamethasone (Alcon Laboratories) eye drops may also assist recovery to some extent.

By measuring the central corneal thickness using AS-OCT, we found that at 1-week post-surgery, corneal edema was the most severe in the ‘R to M’ group. The corneal thickness of all groups was not significantly changed at 1 month after surgery. In addition, the grafts significantly thickened the host cornea without any graft dissolution or dislodgement throughout the study. The stability of the grafts clearly depended on both the tidiness of the femtosecond laser cutting procedure and the implant biocompatibility. Compared with common LK, blade cutting in surgery cannot achieve a consistent depth and thickness on every occasion inside the corneal stroma, and the process is time-consuming. The femtosecond laser is therefore a valuable technique in corneal implantation that substantially reduces surgical time and allows donor and recipient tissues to be precisely matched. This represents a substantial improvement in corneal implantation.

Both human and rabbit grafts were thicker than monkey grafts (81-μm thickness), which might have caused the varying severity of corneal edema and might be related to xenogeneic stress responses. In addition, errors in instrument measurements and dehydration-related changes in the structure of the graft may also have influenced the results. Furthermore, the grafts may have differed in the degree of post-surgical deformation in the anterior and posterior. Refractive diopter corrections and changes in corneal biomechanics should be further studied.

Tear fluid contains large amounts of cytokines, and differences in tear fluid composition reflect the general condition of the ocular surface [30, 31]. IL-1β is mainly involved in inflammatory responses and induces the expression of other inflammatory mediators, such as IL-6 and IL-17 [32]. IL-17 has also been implicated in disrupting the integrity of the corneal barrier [33]. IL-1β, IL-6 and IL-17 have been shown to be inflammatory response factors. Early after implantation, inflammatory responses were the most severe in the “R to M” group and were also observed by clinical observation. The inflammatory responses were lower at 1 month after surgery, while no obvious inflammatory responses were observed in the allotransplantation group throughout the study. Host monkeys in the allotransplantation group exhibited more biological tolerance than was observed in the xenotransplantation group. Additionally, the inflammatory responses to nonhuman xenogenic grafts were most severe within 1 week post-surgery. The severity of inflammatory responses may be related to the differences between species. Furthermore, the expression of most inflammatory response factors was lower at 1-month post-surgery, and this may be associated with the minimally invasive surgical approach. The ability to perform precise cutting allowed the surgeon to perfectly match the ‘stromal pocket’ to the graft, and this might have accelerated the corneal recovery process.

In addition, IL-6 plays an important role in regulatory functions in T cells and B cells, indicating that it has important roles in the immunoregulation of autoimmune diseases and inflammatory responses [34]. The down-regulation of IL-1β and IL-6 expression induced immune tolerance and immunosuppression [35], and the low levels of IL-1β expression observed in the xenotransplantation groups might have contributed to the absence of xenotransplantation rejection.

The TGF-β1 can be activated by inflammatory mediators [32]. TGF-β1 also plays important roles in inflammatory responses and wound repair [36]. The level of TGF-β1 was elevated within 1 week of the surgery, and corneal self-recovery may already have been initiated by this timepoint. In addition, the level of TGF-β1 declined at 1 month after surgery. This decline prevented corneal scarring, which can be caused by excessive TGF-β1 expression, confirming that the grafts and surgical approach used in this study did not disrupt the balance in corneal pathophysiology. In our previous studies [37], changes in IL-1 and TGF-β1 expression were observed in patients who underwent femtosecond laser refractive surgery, and these changes were approximately similar to the changes observed in the present study. SMILE is always associated with improved postoperative comfort [38]. However, we were interested to find that the short-term inflammatory responses observed following corneal intrastromal implantation in this study might have been associated to some extent with lower postoperative discomfort.

However, the changes in the expression patterns of TNF-α and IFN-γ that were observed in the present study were inconsistent with the changes observed in our previous refractive surgery study [37]. These two mediators might be associated with the suppression of corneal rejection. TNF-α is a pleiotropic cytokine that is involved in promoting inflammatory responses, cell apoptosis and necrosis [39]. CD4^+^T cell-mediated delayed hypersensitivity is also a predominant contributor to corneal transplantation immunity [40]. King et al. [40] noted that IL-2, IFN-γ and TNF-α secreted by CD4^+^T cells in corneal allotransplantation rejection. TNF-α and IFN-γ also could activate macrophages and induce inflammatory responses [41]. Both xenotransplantation groups exhibited downtrend expression of TNF-α and IFN-γ, that were observed differently in the allotransplantation group, and this might have been caused by the effects of regulatory T cells (Tregs). Tregs are a subset of T cells and are capable of inhibiting the activation and proliferation of other immunocytes [42]. Additionally, Tregs are involved in immune escape [42]. In the presence of some antigens and inflammatory mediators, including TGF-β and IL-10, CD4^+^T cells can be stimulated to induce Tregs [42]. Similar trends were observed in the expression levels of inflammatory mediators between the glycerol-dehydrated human and rabbit grafts, indicating that the same antigens were primed to activate T cells after implantation. This provides a basis for further studies aimed at investigating the mechanisms underlying the suppression of immunological rejection.

The present study is not without limitations. This is a preliminary study, and we did not test keratocytes repopulation in the grafts. Additionally, whether fresh xenogenic grafts can be used as inlays requires further study. The additive implantation can change the refractive power of the eye by altering the curvature of the cornea or the refractive index of the material itself. Thus, this procedure is potentially useful for treating presbyopia and hyperpresbyopia. Because corneal thickness and volume are positively associated with corneal viscosity and elasticity, we deduced that the corneal stiffness will postoperatively increase. This surgical procedure could also be used in keratoconus and corneal ectasia. This additive surgical procedure offers several advantages over currently used implantation surgical techniques, paramount of which is that the implant may be removed, giving the procedure the potential to be adjustable and reversible.

## Conclusion

Our study showed that glycerol-dehydrated corneal grafts maintained transparency. Following surgery, all of the monkeys survived and exhibited no implant rejection or any adverse complications. Both the allotransplantation and the xenotransplantation grafts remained transparent and elicited minimal inflammatory responses. Furthermore, the inlay lamellae stably matched the host tissues. Glycerol-dehydrated corneal lamellae might have potential use as an alternative inlay xenogeneic material. In addition, femtosecond laser-assisted intrastromal transplantation minimizes the invasiveness and improves the surgical efficiency of this procedure. The host corneas also maintained a high degree of biocompatibility. Hence, in this study, we provide a new treatment for presbyopia, hyperpresbyopia, keratoconus, corneal ectasia, and other diseases. In the future, we will also conduct further studies of potential xenogeneic immune mechanisms and the changes that occur in corneal refraction states after implantation with the aim of creating a foundation for future clinical applications.

## Abbreviations

AS-OCT: Anterior segment optical coherence tomography
FCT: Factual change in central corneal thickness
IFN-γ: Interferon-γ
IL-17: Interleukin-17
IL-1β: Interleukin-1β
IL-6: Interleukin-6
LK: Lamellar keratoplasty
*LSD-t*: Least significance difference test
PCT: Predictable change in central corneal thickness
PKP: Penetrating keratoplasty
PRK: Photorefractive keratectomy
RST: Residual stromal thickness
SMILE: Small incision lenticule extraction
TGF-β1: Transforming growth factor-β1
TNF-α: Tumour necrosis factor-α
